# Integrative multi-omics deciphers the potential mechanism and microbial biomarkers for lymph node metastasis in colorectal cancer

**DOI:** 10.1038/s41598-025-22350-2

**Published:** 2025-11-04

**Authors:** Min Seob Kwak, Jae Myung Cha, Chang Woo Kim, Kyu Yeoun Won, Chang-Il Hwang

**Affiliations:** 1 Department of Internal Medicine, Kyung Hee University Hospital at Gangdong, Kyung Hee University College of Medicine, 892 Dongnam-ro, Gandong-gu, Seoul, 05278 Republic of Korea; 2 Department of Colorectal Surgery, Ajou University School of Medicine, Suwon, Republic of Korea; 3 Department of Pathology, Kyung Hee University Hospital at Gangdong, College of Medicine, Kyung Hee University, Seoul, Republic of Korea; 4 Department of Microbiology and Molecular Genetics, College of Biological Sciences, University of California Davis, Davis, CA 95616 USA

**Keywords:** Multi-omics, Colorectal cancer, Biomarker, Metastasis, Microbiome, Gastrointestinal cancer, Tumour biomarkers

## Abstract

Understanding and accurate diagnosis of lymph node metastasis (LNM) for patients with colorectal cancer (CRC) is essential to determine treatment and follow-up strategies. Therefore, in this study, we aimed to elucidate the biological process and identify the potential biomarker for LNM in CRC.

A total of 30 patients who received a histologically confirmed diagnosis of CRC with Stage I to III and a curative surgery between November 2020 and July 2021 at Kyung Hee university hospital at Gangdong were included. We performed multi-omics approach integrating the data on somatic mutation, transcriptomic expression, DNA methylation, and microbiome with tumor and adjacent matched normal tissues of each patient. In total, 12 significant DEGs between the patients with and without LNM were identified, consisting of significantly upregulated S100A8 gene, a proinflammatory gene. The GSEA revealed that gene sets involving “MULTI CANCER INVASIVENESS” in terms related to epithelial-mesenchymal transition was significantly upregulated in the patients with LNM. Integrated functional analysis of DNA methylation with transcriptome profile shows that significantly hypomethylated promoters of the genes are enriched for LNM. The *phylum* Proteobacteria, unassigned (*p*_PU) presented significantly higher proportions in cancer tissues from the adjacent normal tissues. Notably, when compared to the patients without LNM, the gut microbiota of those with LNM appears to exhibit a significantly lower abundance of the *p*_PU, indicating its potential as promising biomarker for LNM in CRC. We explained the mechanism of tumor spreading using multi-omics analysis and identified the relevant metagenomic biomarker to predict the LNM in CRC by the recognition of host-microbial interaction, thereby can make the cancer surveillance of the patients more individualized and convincing.

**Supplementary Information:**

The online version contains supplementary material available at 10.1038/s41598-025-22350-2.

Colorectal cancer (CRC) is the second leading cause of cancer-related deaths worldwide^1,2^. The global burden of CRC continues to rise, reflecting ongoing challenges in prevention, diagnosis, and treatment^1,2^. Currently, surgical resection is the only curative treatment for locoregional CRC. However, the increased awareness and the implementation of population-based bowel-screening and surveillance programs around the world have shifted the focus towards detecting precancerous dysplastic lesions and early-stage CRC^3,4^, resulting in the increased interest in local endoscopic treatment options.

It is known that regional lymph node metastasis (LNM) is the most significant prognostic factor for tumor aggressiveness and poor prognosis in CRC patients. Therefore, accurate nodal staging is critical at the initial diagnosis of CRC^5^. Furthermore, the presence of LNM is a crucial determinant in further management decisions for both colorectal surgeons and gastroenterologists. For patients without evidence of LNM, complete removal of cancer can be achieved if the primary tumor is completely removed with endoscopic or surgical resection.

Conversely, the adjuvant chemotherapy should be recommended for CRC patients with LNM after surgical resection to eliminate micro-metastasis and to prevent the spread of tumor cells to distant organs^6,7^. Furthermore, additional surgery should be considered for endoscopically treated patients with inaccessible assessment of lymph node status if histological signs after endoscopic resection indicate a high risk of LNM^8^.

Unfortunately, the current diagnosis of LNM still has limitations. Initially, the lymph node in question must be included in the surgical resection, followed by its identification in the pathologic specimen for further evaluation. The metastasis within the lymph node is then detected through meticulous histologic sectioning and pathology review. These processes are not only time-consuming but also rely heavily on the expertise of the pathologist, leading to considerable variability in diagnostic outcomes^9^. The 20% − 30% recurrence rate in tumors is attributed to this where tumors were presumed to be completely excised but lacked visible LNM, likely due to occult lymph node disease^10,11^. The presence of cancer cells in the lymph nodes, serving as effective gateways to the systemic circulation, increasing the risk of recurrence and distant metastasis^12,13^. Improved staging of LNM could potentially identify CRC patients who would benefit from additional adjuvant treatments, thereby improving their prognosis and overall management.

To this end, we aim to enhance the diagnostic accuracy and prognostic assessment of CRC by employing comprehensive multi-omics profiling approaches. We have conducted a detailed analysis of CRC patients, both with and without regional LNM with whole exome sequencing (WES), RNA-seq and DNA methylation and 16 S metagenomics in CRC progression. This multi-omics approach offers a better understanding of the molecular mechanisms of LNM in CRC. At the same time, it facilitates the development of novel diagnostic and prognostic tools that could significantly impact the management and outcomes of CRC patient.

## Materials and methods

### Eligibility criteria and tissue preparation

All patients between 18 and 85 years old who received a histologically confirmed diagnosis of CRC with Stage I to III and a curative surgery between November 2020 and July 2021 at Kyung Hee university hospital at Gangdong were included. The patients who had history of a prior malignancy including CRC, had an inadequate liver and renal function, had a systemic inflammatory disease or chronic inflammatory bowel disease, had any diagnosis of hereditary colorectal cancer syndrome, such as familial adenomatous polyposis or Lynch syndrome, or clinically significant cardiovascular disease were excluded from the study. Tumor and adjacent matched normal tissues were harvested following surgical resection and snap frozen in liquid nitrogen. According to the current official guidelines, at least 14 lymph nodes were sampled from the CRC patients and the specimen findings were reviewed thoroughly by the expert pathologist. We divided patients into two groups according to whether the tumor cells are isolated in lymph node (LNM positive vs. negative). This study was evaluated and approved by the Institutional Review Board of the Kyung Hee University Hospital at Gangdong, Seoul, Republic of Korea (KHNMC IRB No. 2020-09-025). Written informed consent was obtained from all patients before participating in this study.

### WES

Sequencing libraries were generated using the SureSelect Human All Exon V8 kit for sequencing on the Illumina Hiseq 2500 platform. After quality control, sequenced data were aligned to the hg38 (GRCh38) reference genome using the Burrows-Wheeler Aligner. To provide high-quality bam files, poor quality and duplicated reads were filtered out using Samtools. For variant calling, VarScan v2.4.5.was performed with the following filter parameters: coverage (minimum 3×), phred base quality (minimum 20), allele frequency (at least 8%), and a *p* value (less than 0.05). The filtered variants are annotated with SnpEff (v5.0).

### RNA sequencing

We performed RNA sequencing on the samples of included patients using SureSelectXT RNA Direct Library Preparation Kit. Using the Illumina Hiseq 2500, the raw data were generated and their Low-quality reads and adapter sequences were trimmed by Trimmomatic v0.38. Using the StringTie v2.2.0, transcripts and genes were assembled from the aligned reads, followed by read alignment and quantification by HISAT v2.2.1. Genes less than 100 counts per million were filtered out and the counts were normalized with calcNormFactors function. Differential gene expression analysis was performed using the edgeR R package. Differential expressed gene (DEG) was considered significant if log2 fold change was ≥ 2 or ≤ − 2, *p* value was < 0.001 and the adjusted *p* value was < 0.05.

### Methyl-capture sequencing

The sequencing library (SureSelectXT Methyl-Seq Reagent Kit) was prepared and bisulfite converted, amplified and a capture enriching for targeted bisulfite-converted DNA fragments was carried out. After amplifying the captured DNA, each capture was sequenced on the Illumina HiSeq2500 system using 100 bp paired-end sequencing. For the preprocessing and analysis, the CpG_Me alignment pipeline (v1.4)^14^, which is based on Trim Galore (v0.6.5), FastQ Screen (v0.14.0), Bismark (v0.22.3), Picard (v2.18.4), and MultiQC (v1.9), was used to trim adapters and methylation bias, screen for contaminating genomes, align to the reference genome (GRCh38), remove duplicates, calculate coverage and insert size metrics, extract CpG methylation values, generate genome-wide cytosine reports (CpG count matrices), and examine quality control metrics. Differentially methylated region (DMR) calling and most downstream analyses and visualizations were performed via DMRichR (v1.7.8)^14^. To find enriched TF motifs in DMRs, the genomic coordinates of the DMRs from CMB subtype analyses were subjected to HOMER findMotifsGenome.pl.

### Bacterial 16 S ribosomal RNA (rRNA) sequencing

The bacterial 16 S rRNA libraries, directionally targeting the V3-V4 hypervariable region of the bacterial 16 S rRNA gene were prepared and generated data on an Illumina HiSeq. The paired-end sequencing data were analyzed using the Quantitative Insights into Microbial Ecology 2 (QIIME 2) platform. After quality control and denoising of data, we obtained the reads with amplicon sequence variants and their taxonomic assignment was performed based on the SILVA 16 S rRNA database (v.138.1). For basic measures, the α-diversity, including estimates of richness (Ace, Observe, and Chao1) and biodiversity (Pielou, Shanon, and Simpson), and the β-diversity were calculated based on the rooted phylogenetic tree.

### Functional analysis

We measured the functional association between a set of genes and a phenotype in gene expression profiling of CRC patients using the gene set enrichment analysis (GSEA) (GSEA v4.01.0 software). For the analysis, the curated gene sets (c2.all.v2024.1.Hs.symbols.gmt) database, with 1,000 number of permutations, and GSEA false discovery rate < 0.05 was considered statistically significant. To identify functional categories of DEGs, Gene Oncology (GO) enrichment analysis was performed using the Database for Annotation, Visualization and Integrated Discovery. Enriched GO terms were selected with the *p* value less than 0.05.

### Statistical analysis

An independent t-test or Wilcoxon rank-sum test was used to detect differences in baseline characteristics, depending on normality of the data distribution. Differences in distribution of categorical parameters between the groups at baseline were tested by Fisher’s exact tests. All *p* values were two-tailed and *p* values of 0.05 or less were considered to be statistically significant. The data processing and analyses were performed using R (version 4.3.3) and Python (version 3.9.1) scripts. The analyses were run on a server with a 2 × Six-Core Intel Xeon processor, two-GPU Nvidia TITAN X, and 128 GB memory.

## Results

### Baseline characteristics of CRC patients

A total of 33 CRC patients were enrolled in this study. Among them, three patients were excluded because of the poor quality for next-generation sequencing. Of the remaining 30 CRC patient, 16 CRC patients (53%) presented LNM, confirmed by histopathology analysis. Demographics and baseline characteristics were similar in the two groups, LMN-positive and LMN-negative (Table 1). Most patients were older than 65 years of age. The LNM-negative group had relatively more female patients compared to the LNM-positive group. There were no statistical differences between the groups with regard to weight and height. The mean (SD) baseline BMI was 22.3 (3.4) for the patients without LNM and 23.3 (4.1) for those without LNM (*p* = 0.458, Table 1). The LNM-positive patients had a higher ASA score of III, although it did not reach a statistical significance (*p* = 0.517, Table 1). The mean (SD) baseline serum CEA and CA19-9 levels for the LMN-positive group were 14.2 ng/mL (15.7) and 16.8 U/mL (32.6), respectively, which is relatively higher than LNM-negative group (Table 1). The tumor size was similar and the most tumors were located at the sigmoid colon in both groups. The most common histologic type (n [%]) in the primary tumor was moderately differentiated adenocarcinoma (LNM-negative 12 [85.7%]) and -positive (15 [93.8%], respectively). The baseline characteristics of the two patient groups revealed no statistically significant differences (Table 1).

**Table 1:** Baseline characteristics of the patients and tumors included in the study.

		LN metastasis (−)	LN metastasis (+)	*p* value
		(*N* = 14)	(*N* = 16)	
*Patients*				
Age, yr, mean ± SD		66.4 (11.0)	68.8 (12.1)	0.565
*Sex, (%)*				0.980
Female		8 (57.1%)	8 (50.0%)	
Male		6 (42.9%)	8 (50.0%)	
Weight, kg, mean ± SD		56.6 (11.0)	61.1 (14.9)	0.359
Height, cm, mean ± SD		157 (8.8)	160 (10.2)	0.394
BMI, mean ± SD		22.3 (3.4)	23.3 (4.1)	0.458
*ASA classification, (%)*				0.517
1		3 (21.4%)	3 (18.8%)	
2		7 (50.0%)	5 (31.2%)	
3		4 (28.6%)	8 (50.0%)	
CEA, ng/mL, mean ± SD		5.0 (11.9)	14.2 (15.7)	0.078
CA19-9, U/mL, mean ± SD		6.0 (5.5)	16.8 (32.6)	0.213
*Tumors*				
Maximal tumor size, mean ± SD		4.4 (2.7)	5.0 (1.9)	0.517
*Location, (%)*				0.886
CE		2 (14.3%)	1 (6.3%)	
AC		1 (7.1%)	4 (25.0%)	
HF		2 (14.3%)	1 (6.3%)	
TC		1 (7.1%)	1 (6.3%)	
DC		0 (0.00%)	1 (6.3%)	
SC		5 (35.8%)	5 (31.1%)	
RSJ		2 (14.3%)	3 (18.7%)	
RE		1 (7.1%)	0 (0.00%)	
*Histology, (%)*				0.209
Adenocarcinoma, WD		2 (14.3%)	0 (0.00%)	
Adenocarcinoma, MD		12 (85.7%)	15 (93.8%)	
Signet ring cell carcinoma		0 (0.0)	1 (6.2)	

SD, standard deviation; BMI, body mass index; ASA, American society of anesthesiologists; CEA, cancer embryonic antigen; CA19–9, cancer antigen 19–9; CE, cecum; AC, ascending colon; HF, hepatic flexure; TC, transverse colon; DC, descending colon; SC, sigmoid colon; RSJ, rectosigmoid junction; RE, rectum; WD, well differentiated; MD, moderately differentiated; PD, poorly differentiated; LN, lymph node.

### Mutational landscape of CRC

**Fig. 1:**
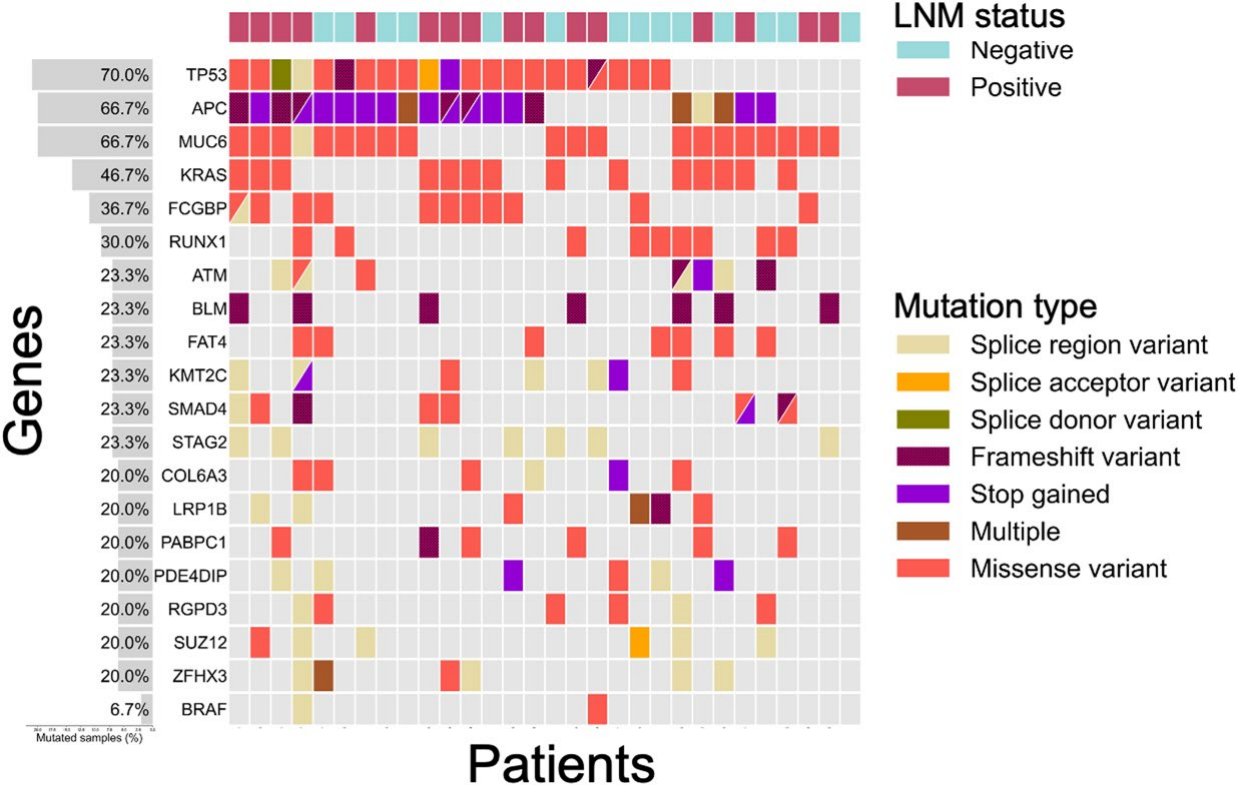
Somatic mutational profiles in CRC patients. Somatic mutations were identified by whole exome sequencing. The top 19 genes selected based on a recurrence frequency > 20% across the cohort. The BRAF gene was additionally included, despite its mutation frequency being below the 20% threshold. CRC, colorectal cancer.

To investigate the mutational spectrum in CRC, we performed exome capture DNA sequencing on each tumor tissue and the paired adjacent normal tissue from 30 patients. After exclusion of the germline mutations and low impact severity, the WES from 30 patients with CRC revealed a total of 32,627 variants. Additionally, the variants of uncertain significance were filtered out, such as non-coding transcript exon variant, intron variant, synonymous variant, disruptive in-frame deletion, 5’-UTR variant, 3’-UTR variant, downstream gene variant, upstream gene variant. Finally, 376 mutated genes were identified based on the cancer driver genes (Supplementary Table S1). Among them, we focused on the 19 most recurrently mutated genes (*TP53*, *APC*, *MUC6*, *KRAS*, *FCGBP*, *RUNX1*, *ATM*, *BLM*, *FAT4*, *KMT2C*, *SMAD4*, *STAG2*, *COL6A3*, *LRP1B*, *PABPC1*, *PDE4DIP*, *RGPD3*, *SUZ12*, and *ZFHX3*, given in decreasing order of mutation rate) and the manually added CRC-related gene (*BRAF*) (Fig. 1).

Consistent with the other observations in CRC, we also identified the three most frequently mutated genes in CRC: *TP53*, *APC*, and *KRAS*. In comparison with the mutation frequencies reported by TCGA CRC dataset, the mutation frequencies of *TP53*, *APC*, and *KRAS* genes were higher in our patients (Fig. 1). Among them, the prevalence of *KRAS* mutations was; codon 12, 11 patients (78.6%), specifically 8 patients, c.35 G > A (G12D), 3 patients, c.35 G > T (G12V); codon 13, 2 pts (14.3%), c.38 G > A (G13D), and; codon 61, 1 patient (7.1%), c.183 A > C (Q61H) (Supplementary Fig. S1). Among the *TP53* mutations, 17 (81.0%) missense mutations were present in the DNA binding domain, and one (36%) mutation was frameshift mutation in the DNA binding domain (Fig. 1 and Supplementary Table S2).

Intriguingly, we observed relatively higher prevalence of non-silent mutations in *MUC6* (66.7%), *FCGBP* (36.7%), *RUNX1* (30.0%), *ATM* (23.3%), and *BLM* (23.3%), compared with previous literatures. Mutations in the *BRAF* gene occurred at a low frequency of 6.7% of the patients (1 patient, c.1799T > A (p.V600E); 1 patient, splice region variant), mutually exclusive to *KRAS* mutations (Supplementary Table S2). While we attempted to identify LNM-specific mutation profiles, there were no significant differences in mutation rates among the top 3 CRC driver genes, *TP53*, *APC* and *KRAS* (*p* = 0.811, *p* = 0.518 and *p* = 0.981, respectively). No significant mutational landscape between LNM-positive and -negative samples prompted us to investigate transcriptomes and epigenomes.

### Gene expression signature in CRC patients

**Fig. 2:**
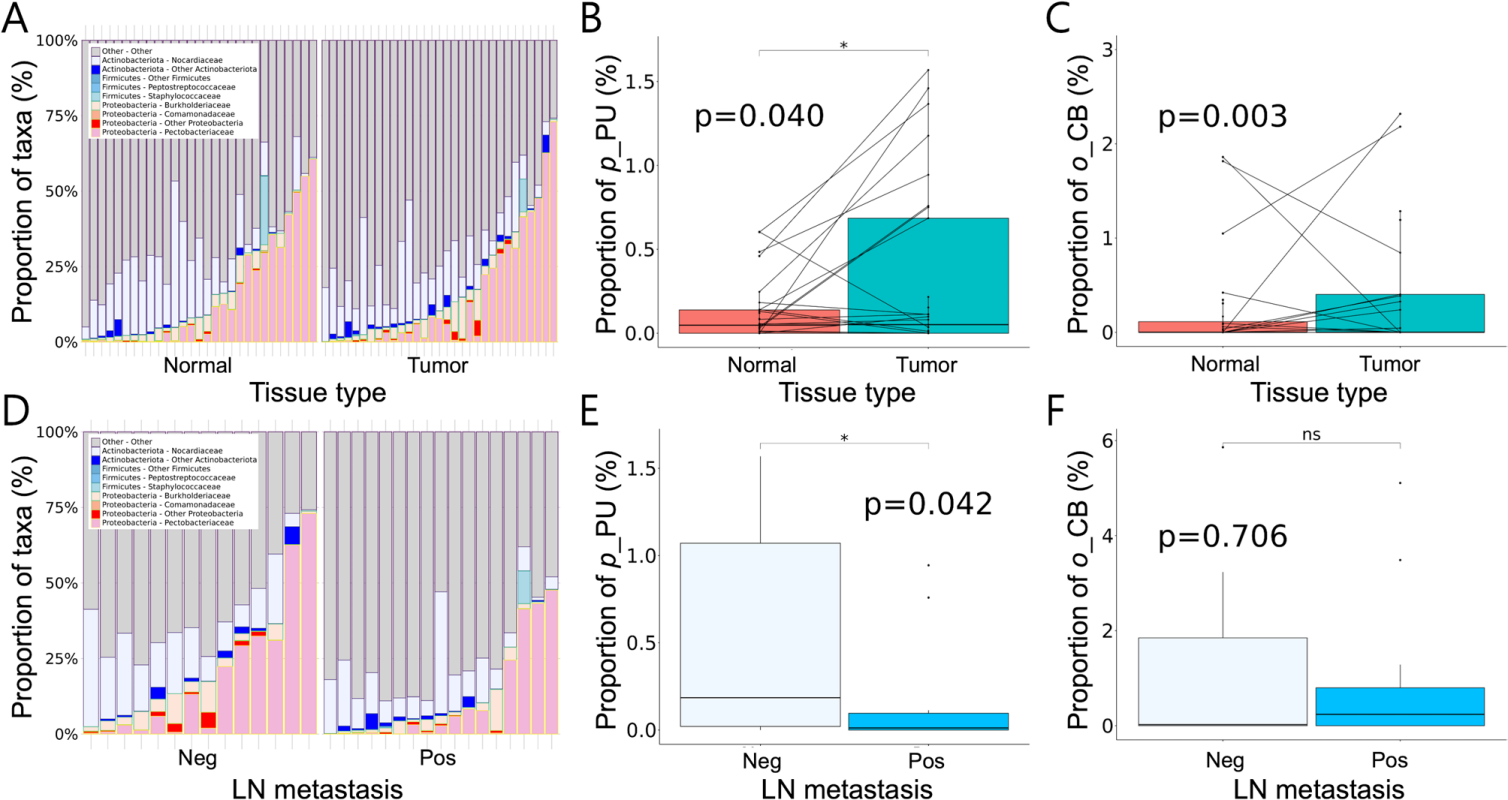
Volcano plots and PCA plots of differentially expressed genes (DEGs). Volcano plot (**A**), PCA plot (**B**), and GSEA analysis (**C**), in of DEGs between tumor and matched normal tissues in CRC patients; Volcano plot (**D**), PCA plot (**E**), and GSEA analysis (**F**) in of DEGs between the CRC patients with- and without LNM. Volcano plot; Red dots indicate significantly up- and down-regulated genes (Benjamini-Hochberg algorithm correction, *p* value < 0.001, absolute log_2_FC higher than 2), blue dots represent the genes with *p* value < 0.001 but not log_2_FC cutoff and grey dots show non-significantly altered genes, which are not meeting the criteria mentioned above, between the two groups). GSEA; the x-axis shows the DEGs belonging to each pathway and the y-axis shows positive/negative ES for up-/down-regulated genes associated with the pathway. PCA, principal component analysis; DEGs, differentially expressed genes; GSEA, Gene set enrichment analysis; CRC, colorectal cancer; LNM, lymph node metastasis; FC, fold change; ES, enrichment scores.

To identify genes and pathways responsible for CRC progression and metastasis, we performed RNA-seq analysis for the CRC primary tumors from both LMN-positive and -negative as well as the adjacent normal tissues. First, we attempted to identify the DEGs specific to CRC compared to the adjacent normal tissues, resulting in the identification of 201 statistically significant DEGs (32 up-regulated genes and 169 down-regulated genes) (Fig. 2A and Supplementary Table S3). The principal component analysis (PCA) using the whole transcriptome gene expression profiles showed clear segregation of samples based on the diagnosis of CRC (tumor vs. normal) (Fig. 2B). To further characterize the pathways altered in CRC progression, we performed the gene set enrichment analysis (GSEA) and gene ontology (GO) analysis. The GSEA revealed that the gene set involving “COLON AND RECTAL CANCER UP” was significantly up-regulated in CRC primary tumor tissues compared to the adjacent normal tissues (Fig. 2C and Supplementary Fig. S2A). Additionally, GO analysis indicated that numerous development-related terms were more prevalent in CRC tumors than in adjacent normal tissues (Supplementary Fig. S2C).

Likewise, in comparison between LNM-positive and -negative samples, we identified 12 significant DEGs, including 2 upregulated genes (*HCAR2* and *S100A8*) and 10 down-regulated genes (*AQP8*, *H19*, *RNU5B-1*, *SNORA61*, *SNORA68*, *SNORA71D*, *SNORA74B*, *SNORA9*, *SNORD3A*, and *SNORD3C*) (Fig. 2D and Supplementary Table S4), while the PCA displayed less pronounced segregation based on the LNM status (Fig. 2E). This suggests that although the transcriptomic differences between cancerous and normal tissues were obvious, the distinctions between LMN-positive and -negative are more subtle. Notably, we found that “MULTI CANCER INVASIVENESS” gene signature was markedly enriched in the CRC tumors with LNM compared to LMN-negative samples in the GSEA (Fig. 2F and Supplementary Fig. S2B), suggesting that unique transcriptome changes responsible for CRC progression and metastasis are highly enriched in LMN-positive compared to LMN-negative tumors. Furthermore, GO analysis revealed that neurodevelopment-related terms were prominently represented in LNM-positive compared to LNM-negative tumors, highlighting distinct biological pathways potentially involved in metastatic progression (Supplementary Fig. S2D).

### Integrative analysis of DNA methylation and gene expression in CRC patients

**Fig. 3:**
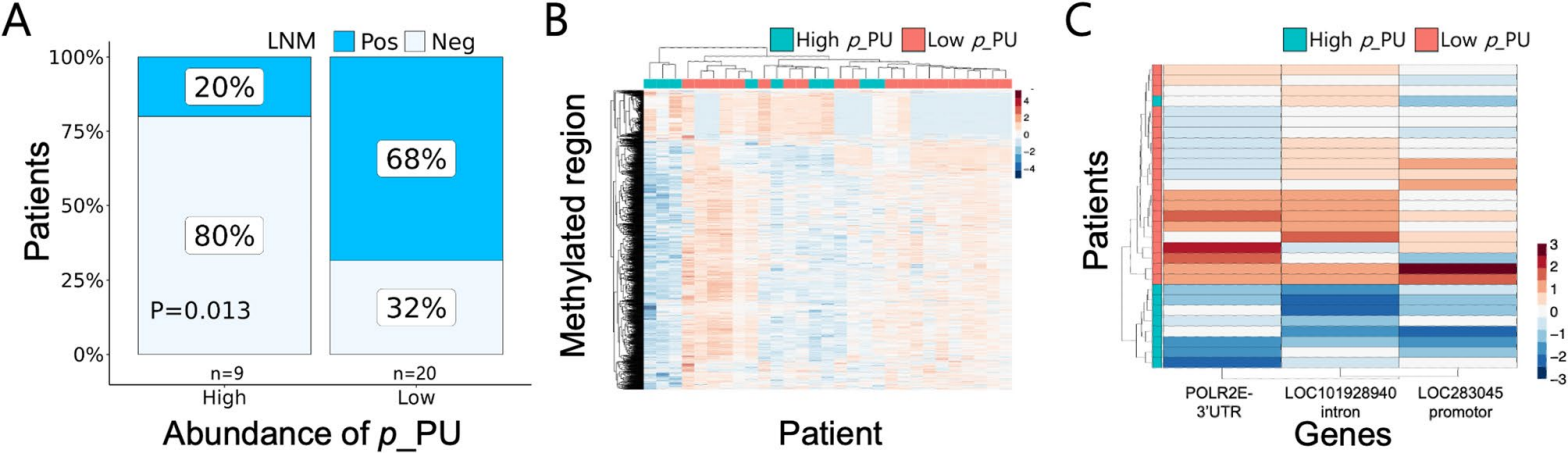
PCA plot and heatmap based on the differentially methylated regions. PCA plot (**A**), hierarchical clustering and heatmap (**C**) demonstrates separation between normal and CRC tissue samples. PCA (**B**) fails to identify significant separation between positive vs. negative LNM, while hierarchical clustering and heatmap (**D**) represents distinct DNA methylation patterns based on methylated fraction between the groups. The enrichment plot with gene sets having DMR in their promoter regions showed significant enrichment in tumors vs. matched normal tissues (**E**); in positive LNM vs. negative LNM (**F**). PCA, principal component analysis; CRC, colorectal cancer; LNM, lymph node metastasis; DNA, deoxyribonucleic acid; DMR, differentially methylated region.

Next, we reasoned that distinct transcriptome profiles in CRC progression could be dictated by epigenetic alterations such as DNA methylation. To address this, we performed methyl-capture sequencing on the same tumor and adjacent normal tissues that were used for the RNA-seq analysis, allowing for base-pair resolution of genome-wide DNA methylation. PCA of the global DNA methylation patterns revealed a distinct DNA methylation landscape between CRC tumors and the matched adjacent normal tissues, mirrored by distinct transcriptome profiles (Fig. 3A and Supplementary Fig. S3A). While we did not see clearly distinct clusters between LMN-positive and -negative similar to the transcriptome profiles (Fig. 3B and Supplementary Fig. S3B), we found that LMN-positive tumors tend to have more heterogeneous DNA methylation than LMN-negative, suggesting that CRC samples might undergo epigenetic alterations in various genes and pathways during CRC progression and metastasis. Using the DMRichR package, we identified the differentially methylated regions (DMRs) between tumor vs. adjacent normal and between LMN-positive vs. -negative. From this analysis, we were able to identify 43,968 CRC-specific DMRs (6,348 hypermethylated and 37,620 hypomethylated regions) compared to the adjacent normal tissues and 131 LNM-specific DMRs (91 hyper-methylated and 40 hypo-methylated) compared to LMN-negative samples (Fig. 3C and D). To address whether these DMRs are associated with the differential expression, we identified the genes associated with DMRs using the GREAT and asked whether these genes are differentially expressed using the GSEA. The analysis revealed that the genes associated with hypermethylated regions in CRC-specific context were down-regulated (Fig. 3E). Likewise, the genes associated with hypomethylated regions in LMN-specific context were up-regulated in LMN-positive samples (Fig. 3F), confirming the inverse correlation between DNA methylation and the associated genes.

Recent analyses of the transcriptomic profiles of CRC stratified CRC into 4 distinct molecular subtypes with distinct biological and clinical features^15^. The close relation between gene expression profiles and DNA methylation patterns prompted us to explore whether these molecular subtypes could be determined by methylation data. To this end, we classified the CRC patients into 4 consensus molecular subtypes (CMS) based on gene expression profiling, resulting in transcriptionally distinct 4 cm groups: CMS1 (*n* = 2), CMS2 (*n* = 8), CMS3 (*n* = 10), and CMS4 (*n* = 7), except the 3 patients who were not assigned to any CMS. (Supplementary Fig. S4).

Based on the stratification, we identified unique DMRs for each CMS subtype (Supplementary Fig. S4, Supplementary Table S5, S6, S7 and S8) and these DMRs were subjected to the HOMER motif analysis. Among these, hypomethylated DMRs in CMS2 displayed the enriched transcription factor (TF) binding motif for *HNF* transcription factors (Supplementary Fig. S5). Given the fact that CMS2 is closely associated with *HNF4A* amplifications, it suggests active HNF transcription factor networks might be responsible for maintaining the hypomethylated regions of CMS2. Likewise, we also found the enriched TF binding motifs for TWIST and SMAD transcription factors for the hypomethylated regions in CMS4 (Supplementary Fig. S5). Previously, CMS4 was characterized by up-regulation of EMT and activation of TGF-β signaling. It is tempting to speculate that EMT-TF and TGF-β signaling networks might be responsible for hypomethylated status of these TF binding sites, contributing to EMT phenotypes. These results suggest that subtype-specific signaling programs in CRC may be, at least in part, epigenetically regulated, and that DNA methylation signatures may help reinforce and sustain the transcriptional identity of distinct CMS groups.

### Microbial signatures in CRC

To investigate key features of microbial environments in CRC development and LNM, we performed 16 S-rRNA sequencing analyses on CRC tumors, both LNM-positive and negative, and their paired adjacent normal tissues.

**Fig. 4:**
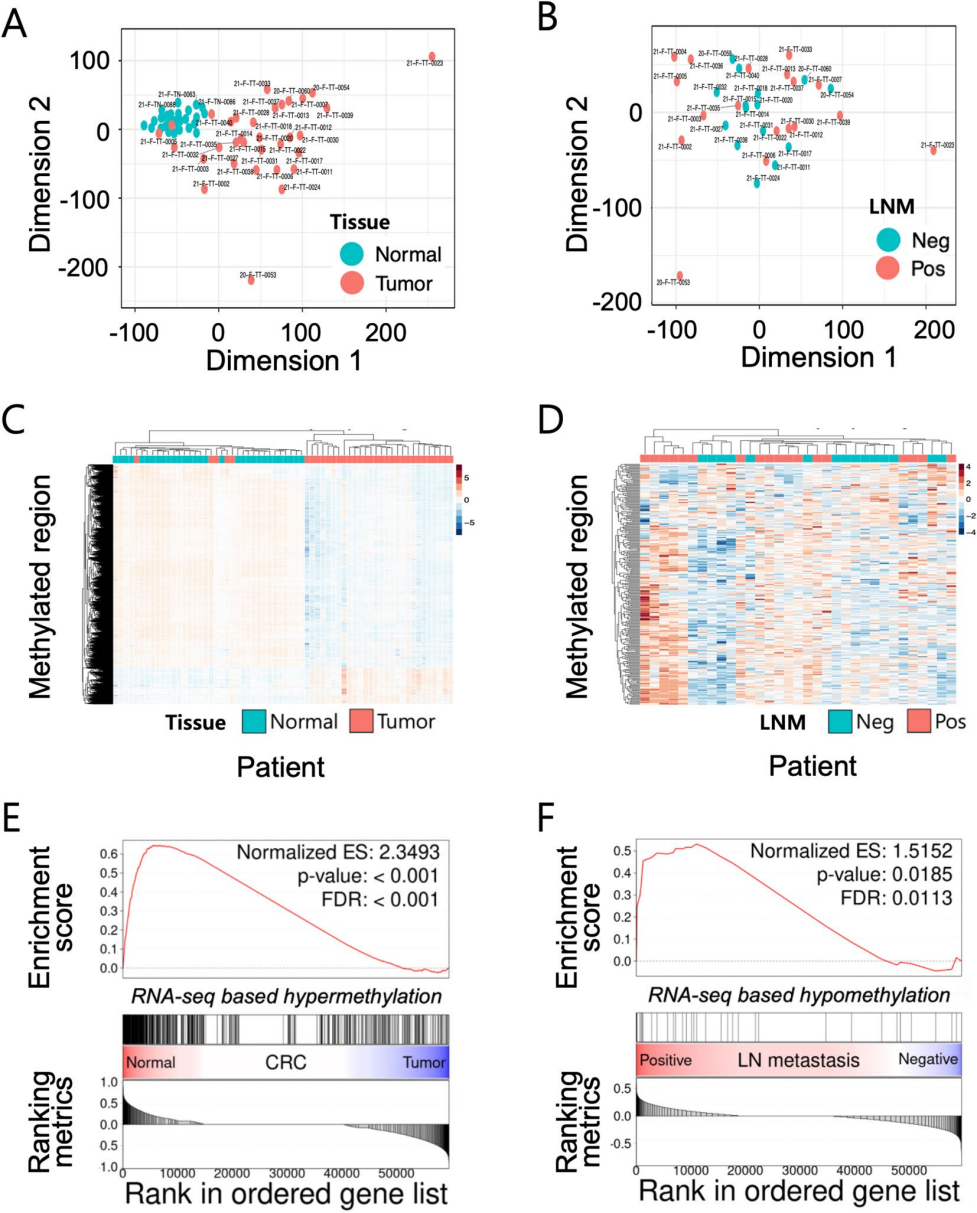
Taxonomy bar plot showing taxonomic composition of microbial communities for each individual sample of the patients with CRC at the level of class. Significant compositional change (**A**) Taxonomy bar plot between normal vs. tumor tissues. (**D**) Taxonomy bar plot between LNM negative vs. positive. Significant compositional differences in *p*_PU were shown between normal and CRC (**B**) tissues; between the patients with- and without LNM (**E**). The composition of *o*_CB at the order level is significantly higher in CRC tissue than in normal tissues (**C**), although the compositional differences between positive and negative LNM did not show any significant differences in *o*_CB (F). CRC, colorectal cancer; LNM, lymph node metastasis; *p*_PU, *phylum* Proteobacteria unassigned; *o*_CB, *order* Corynebacteriales.

**Fig. 5:**
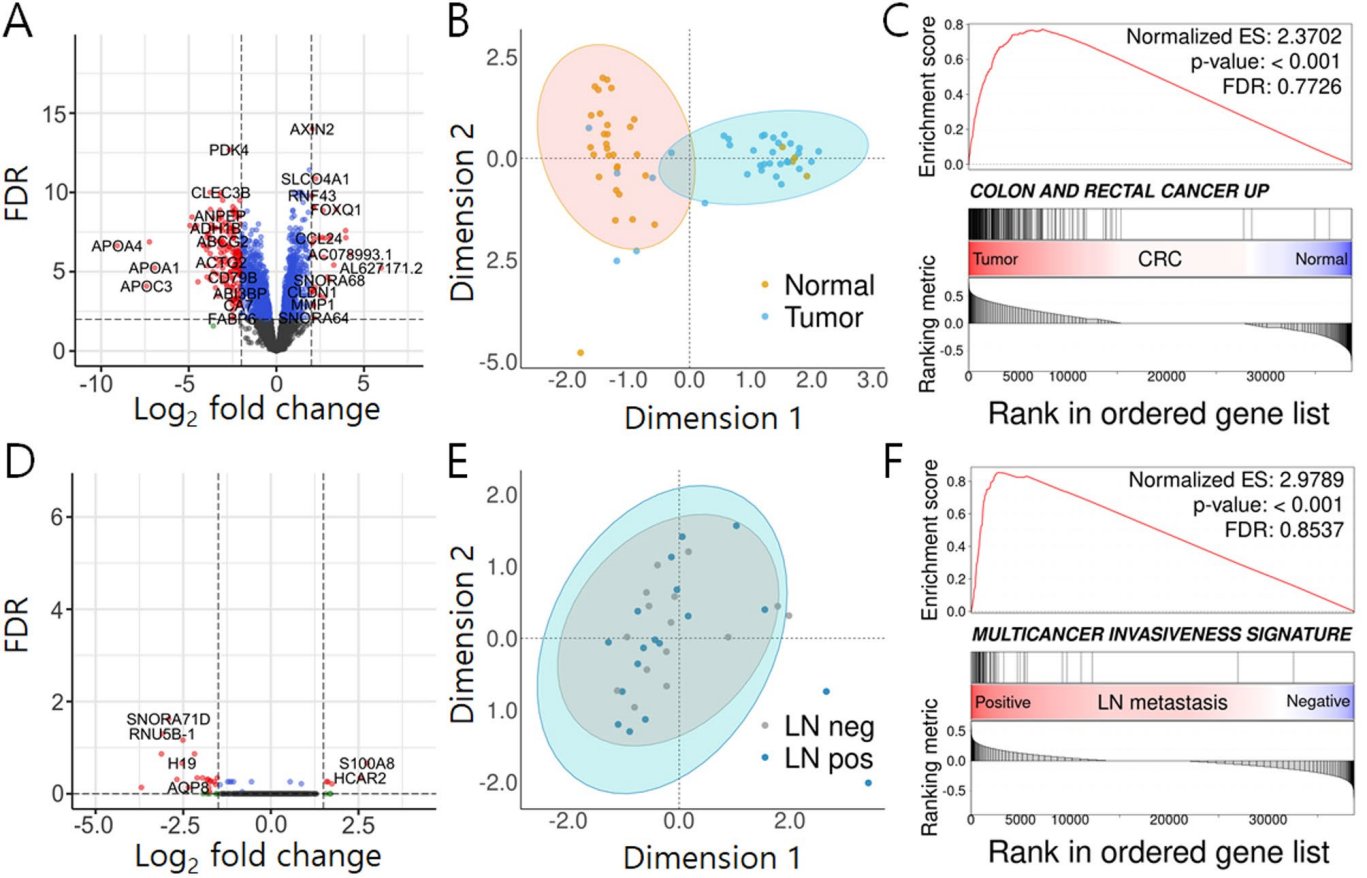
(**A**) The LNM positive rate comparison between low *p*_PU and high *p*_PU groups in patients with CRC, (**B**) Hierarchical clustering and heatmap of DNA methylome between low *p*_PU and high *p*_PU groups in CRC patients. (**C**), By feature selection analysis using machine learning, potential genes were identified to differentiate the *p*_PU from high *p*_PU groups. LNM, lymph node metastasis; *p*_PU, *phylum* Proteobacteria unassigned; CRC, colorectal cancer; DNA, deoxyribonucleic acid.

Strikingly, the *phylum* Proteobacteria, unassigned (*p*_PU) and the *order* Corynebacteriales (*o*_CB) presented significantly higher proportions in tumor tissues compared to the adjacent normal tissues through taxonomy evaluation (*p* = 0.040 and *p* = 0.003, respectively), although the α-diversity and the β-diversity between the groups did not reach statistical significance. (Figure 4A and B, and C). Furthermore, when compared to the patients without LNM, the gut microbiota of those with LNM appeared to exhibit a significantly lower abundance of the *p*_PU, suggesting its potential as a promising biomarker for LNM in CRC (*p* = 0.042) (Fig. 4D and E, and F). This data suggests that *p*_PU could serve as a potential diagnostic/prognostic biomarker for CRC. After excluding the samples without Proteobacteria-related sequences, we divided the CRC patients into two groups –high *p*_PU and low *p*_PU – with a cut-off point at 15% proportion derived from the receiver operating characteristic analysis. The results showed that the CRC patients who have low proportion of *p*_PU are more likely to have LNM (Fig. 5A). Since the LNM status was associated with both *p*_PU enrichment and DNA methylation pattern, we hypothesized that specific DMRs might be associated with epigenetic differences between high *p*_PU and low *p*_PU groups, prompting a further DMR analysis. The DMR analysis resulted in the identification of 1,887 DMRs (1,575 hypermethylated and 312 hypomethylated regions) and the PCA showed relatively distinct separation between the two groups of subjects within two principal components (Supplementary Fig. S6 and Fig. 5B). Given the close association between p_PU and LNM status, we thought that the identification of potential biomarkers to predict the LNM status might be clinically beneficial. To this end, using the machine learning algorithm, we identified three significant differentially methylated genes (Polymerase II subunit E [*POLR2E*] -3’-UTR, *LOC101928940* intron, and *LOC283045* promotor) for discriminating high *p*_PU and low *p*_PU (*p* = 0.020, *p* = 0.022 and *p* = 0.023, respectively) (Fig. 5C). The identification of the 3’-UTR of the POLR2E gene as a differentially methylated region highlights its potential importance, given its previous implications in gastrointestinal and esophageal cancers and its association with increased gene expression^16–19^. Taken together, these findings highlight the potential of using specific metagenomic profiles and epigenetic markers as diagnostic tools to enhance the stratification and management of CRC patients, particularly in identifying those at higher risk for LNM.

## Discussion

Here, we present a comprehensive multi-omics analysis of the mutational landscape, transcriptomic landscape, and epigenetic landscape of DNA methylation, along with metagenomic information to decipher the intricate molecular landscape in metastatic progression of CRC. To the best of our knowledge, this is the first study to describe etiology of host-microbial interactions and to identify a predictive metagenomic biomarker for LNM by integrating multi-omics data in patients with CRC. Currently, many other mutational prognostic markers have been extensively studied owing to genetic alterations that lead to the activation of proto-oncogenes, inactivation of tumor suppressor genes, and abnormalities of DNA repair mechanisms. However, their effectiveness as prognostic tools remain unclear due to their conflicting results. For instance, recent US multi-center study suggests that genetic alteration of *TP53* is associated with progression to metastatic disease in CRC^20^. Meanwhile, two Chinese studies show that *KRAS* and *BRAF* mutations are associated with lymph node or distant metastasis^21,22^, while another Japanese study shows that *SMAD4* mutation in primary tumors occurs in intramucosal carcinoma and more commonly in invasive CRC with distant metastases^23^. In addition, previous large-scale molecular analysis shows the association of lung metastasis and the presence of *NRAS* mutations in CRC^24^. While we were able to identify common driver mutations in *TP53*,* APC*, and *KRAS* genes with similar frequencies (70.0%, 66.7%, 21.5%, and 46.7%, respectively), the analysis for their prognostic values for nodal metastasis in CRC did not exhibit significance. These mutations, while frequently observed in CRC, are likely insufficient on their own to determine metastatic behavior and may act in concert with other factors such as tumor microenvironment, patient-specific characteristics (e.g., age, sex), molecular subtypes, and staging. Moreover, the inconsistency in mutation-based prognostic markers across different studies may reflect technical limitations such as small sample sizes and heterogeneity in patient populations.

It is increasingly recognized that epigenetic alterations and transcriptomic changes may serve as key drivers of CRC progression and metastasis, potentially offering more consistent insights into the mechanisms underlying disease. For instance, one of the genes highly expressed in LNM-positive patients was the S100A8 gene, which has been implicated in enhancing CRC cell migration and invasion, and in activating mucosal CD4 + T cells, thereby mediating Th1 pro-inflammatory responses^25–27^. Furthermore, our GSEA analysis reveals EMT/inflammation signatures and significant enrichment in multi-cancer invasiveness signatures, suggesting that the metastatic spread of cancer cells is promoted by the activation of these signaling cascades, with directional regulation of genes through hypomethylated promoters further elucidating the role of epigenetic modulation in the acquisition of metastatic capabilities. Taken together, these transcriptomic and epigenetic alterations – including DEGs and DMRs- may serve as novel predictive biomarkers for LNM in CRC.

Over the past decade, increasing evidence has suggested that gut microbial communities play a crucial role in the development, progression and metastasis of CRC. This has been attributed to the diverse metabolites produced by gut microbiota, which can be significantly altered by bacterial dysbiosis^28–30^. Our metagenomic analysis shows an evident association of *p*_PU with CRC progression, specifically the presence of LNM is significantly more frequent in the patients who have low *p*_PU. The *phylum* Proteobacteria are the representative bacterial group in CRC, including many known pathogens such as Vibrio cholerae, Salmonella, E. coli, Campylobacter, and Helicobacter pylori as well as unknown free-living species, including many nitrogen-fixing species^31–33^. Recent research involving both Asian and Western patient cohorts demonstrated that Proteobacteria are significantly more abundant in CRC tissues than in normal tissues^31,34^. Consistent with our observation, another study observed a decreasing trend in the composition of Proteobacteria as CRC progresses^35^. Previous studies have established a link between microbial dysbiosis and host immune responses in CRC. For instance, using mouse models it has been shown that changes in gut norepinephrine levels^36^ and inflammation related to IL-10 deficiency^37^ can increase Proteobacteria levels, suggesting that these bacteria may influence CRC development through interactions with the host’s immune system. Our findings suggest that changes in the abundance of Proteobacteria during CRC progression might be related to their roles in the tumor microenvironment, although further investigation is needed to clarify these relationships.

Our study provides new insights into patient risk stratification about the LNM of CRC patients based on the composition of the specific bacteria in the tissue microbiota. Through this biomarker, identifying the patients with apparent benefit to intensive surveillance after endoscopic and/or radical resection of CRC enables earlier recurrence detection or better overall survival for patients in clinical practice. In particular, this finding has clinical value in early-stage CRC patients undergoing local resection, where accurate nodal assessment is not feasible. Integration of such microbial features into clinical workflows may complement histopathologic criteria and refine post-resection management. However, the *p*_PU, unknown members of *phylum* Proteobacteria, that cannot be annotated with referenced biological information should be further characterized for their functional potential. We also acknowledge the lack of ethnic diversity and the result from a single center in present study potentially limits the generalizability of the findings. Further analyses, including shotgun metagenomic sequencing approaches and multi-center, multi-national study, are necessary to address confounding factors effectively. In this regard, we are currently working to expand our investigation through broader collaborative efforts to validate and extend these findings in larger and more diverse CRC cohorts.

In conclusion, we provide the potential mechanism and the relevant metagenomic biomarker of CRC LNM using multi-omics analyses. Our findings contribute to a better understanding of the underlying molecular and microbial interactions in CRC and offer a new approach to patient stratification and management. A more personalized approach for CRC, based on our research, paves the road toward improved individualized cancer surveillance for CRC patients.

## Supplementary Information

Below is the link to the electronic supplementary material.

Supplementary Material 1

Supplementary Material 2

## Data Availability

The datasets analyzed during the current study are available in the Gene Expression Omnibus (GEO), (https://www.ncbi.nlm.nih.gov/geo/query/acc.cgi? acc=GSE308020).

## Abbreviations

CRC: Colorectal cancer
LNM: Lymph node metastasis
GSEA: Gene set enrichment analysis
GO: Gene ontology
CMS: Consensus clustering subtypes
PCA: Principal component analysis
p_PU: Phylum proteobacteria unassigned
o_CB: Order corynebacteriales
DNA: Deoxyribonucleic acid
ACE: Abundance-based coverage estimator
DMR: Differentially methylated regions
FC: Fold change
DEGs: Differentially expressed genes
ES: Enrichment scores
NES: Normalized enrichment score
SD: Standard deviation
BMI: Body mass index
ASA: American society of anesthesiologists
CEA: Cancer embryonic antigen
CA19–9: Cancer antigen 19–9
CE: Cecum
AC: Ascending colon
HF: Hepatic flexure
TC: Transverse colon
DC: Descending colon
SC: Sigmoid colon
RSJ: Rectosigmoid junction
RE: Rectum
WD: Well differentiated
MD: Moderately differentiated
PD: Poorly differentiated
LN: Lymph node

## References

[1] Kratzer, T. B. et al. Cancer statistics for American Indian and Alaska native individuals, 2022: including increasing disparities in early onset colorectal cancer. *CA Cancer J. Clin.* **73**, 120–146. 10.3322/caac.21757 (2023). 36346402 10.3322/caac.21757

[2] Siegel, R. L., Miller, K. D., Fuchs, H. E. & Jemal, A. Cancer statistics, 2022. *CA Cancer J. Clin.* **72**, 7–33. 10.3322/caac.21708 (2022). 35020204 10.3322/caac.21708

[3] Logan, R. F. et al. Outcomes of the bowel cancer screening programme (BCSP) in England after the first 1 million tests. *Gut* **61**, 1439–1446. 10.1136/gutjnl-2011-300843 (2012). 22156981 10.1136/gutjnl-2011-300843 PMC3437782

[4] Inadomi, J. M. Screening for colorectal neoplasia. *N Engl. J. Med.* **376**, 149–156. 10.1056/NEJMcp1512286 (2017). 28076720 10.1056/NEJMcp1512286

[5] Benson, A. B. et al. Colon Cancer, version 3.2024, NCCN clinical practice guidelines in oncology. *J. Natl. Compr. Canc Netw.* **22**10.6004/jnccn.2024.0029 (2024). 10.6004/jnccn.2024.0029 38862008

[6] Gunderson, L. L., Jessup, J. M., Sargent, D. J., Greene, F. L. & Stewart, A. K. Revised TN categorization for colon cancer based on National survival outcomes data. *J. Clin. Oncol.* **28**, 264–271. 10.1200/jco.2009.24.0952 (2010). 19949014 10.1200/JCO.2009.24.0952 PMC2815715

[7] Vogel, J. D., Eskicioglu, C., Weiser, M. R., Feingold, D. L. & Steele, S. R. The American society of colon and rectal surgeons clinical practice guidelines for the treatment of colon cancer. *Dis. Colon Rectum*. **60**, 999–1017. 10.1097/dcr.0000000000000926 (2017). 28891842 10.1097/DCR.0000000000000926

[8] Oka, S. et al. Treatment decision for locally resected T1 colorectal Carcinoma–Verification of the Japanese guideline criteria for additional surgery based on Long-Term clinical outcomes. *Official J. Am. Coll. Gastroenterol. | ACG*. **119**, 2019–2027. 10.14309/ajg.0000000000002715 (2024). 10.14309/ajg.0000000000002715 PMC11288396 38345215

[9] Compton, C. C. & Greene, F. L. The staging of colorectal cancer: 2004 and beyond. *CA Cancer J. Clin.* **54**, 295–308. 10.3322/canjclin.54.6.295 (2004). 15537574 10.3322/canjclin.54.6.295

[10] Hyslop, T., Weinberg, D. S., Schulz, S., Barkun, A. & Waldman, S. A. Occult tumor burden predicts disease recurrence in lymph node-negative colorectal cancer. *Clin. Cancer Res.* **17**, 3293–3303. 10.1158/1078-0432.Ccr-10-3113 (2011). 21307149 10.1158/1078-0432.CCR-10-3113 PMC3096730

[11] Lips, D. J. et al. The influence of micrometastases on prognosis and survival in stage I-II colon cancer patients: the Enroute⊕ study. *BMC Surg.* **11**, 11. 10.1186/1471-2482-11-11 (2011). 21569373 10.1186/1471-2482-11-11 PMC3123166

[12] Brown, M. et al. Lymph node blood vessels provide exit routes for metastatic tumor cell dissemination in mice. *Science* **359**, 1408–1411. 10.1126/science.aal3662 (2018). 29567714 10.1126/science.aal3662

[13] Pereira, E. R. et al. Lymph node metastases can invade local blood vessels, exit the node, and colonize distant organs in mice. *Science* **359**, 1403–1407. 10.1126/science.aal3622 (2018). 29567713 10.1126/science.aal3622 PMC6002772

[14] Laufer, B. I. et al. Placenta and fetal brain share a neurodevelopmental disorder DNA methylation profile in a mouse model of prenatal PCB exposure. *Cell. Rep.* **38**10.1016/j.celrep.2022.110442 (2022). 10.1016/j.celrep.2022.110442 PMC8941983 35235788

[15] Guinney, J. et al. The consensus molecular subtypes of colorectal cancer. *Nat. Med.* **21**, 1350–1356. 10.1038/nm.3967 (2015). 26457759 10.1038/nm.3967 PMC4636487

[16] Zhang, Y. K. et al. The POLR2E rs3787016 polymorphism is associated with susceptibility to and prognosis of gastric cancer. *Neoplasma* **68**, 665–671. 10.4149/neo_2021_201125N1277 (2021). 33847132 10.4149/neo_2021_201125N1277

[17] Kang, M. et al. Long noncoding RNAs POLR2E rs3787016 C/T and HULC rs7763881 A/C polymorphisms are associated with decreased risk of esophageal cancer. *Tumour Biol.* **36**, 6401–6408. 10.1007/s13277-015-3328-z (2015). 25874495 10.1007/s13277-015-3328-z

[18] Baili, E. et al. Genetic impact of HOTAIR, LINC00951, POLR2E and HULC polymorphisms in histopathological and laboratory prognostic factors in esophageal cancer in the west: A Case-Control study. *Cancers* **16**, 537 (2024). 38339289 10.3390/cancers16030537 PMC10854877

[19] McGuire, M. H. et al. Pan-cancer genomic analysis links 3’UTR DNA methylation with increased gene expression in T cells. *EBioMedicine* **43**, 127–137. 10.1016/j.ebiom.2019.04.045 (2019). 31056473 10.1016/j.ebiom.2019.04.045 PMC6558231

[20] Yaeger, R. et al. Clinical sequencing defines the genomic landscape of metastatic colorectal cancer. *Cancer Cell.* **33**, 125–136e123. 10.1016/j.ccell.2017.12.004 (2018). 29316426 10.1016/j.ccell.2017.12.004 PMC5765991

[21] Li, W. et al. Colorectal carcinomas with KRAS codon 12 mutation are associated with more advanced tumor stages. *BMC Cancer*. **15**, 340. 10.1186/s12885-015-1345-3 (2015). 25929517 10.1186/s12885-015-1345-3 PMC4423107

[22] Chen, J. et al. BRAF V600E mutation and KRAS codon 13 mutations predict poor survival in Chinese colorectal cancer patients. *BMC Cancer*. **14**, 802. 10.1186/1471-2407-14-802 (2014). 25367198 10.1186/1471-2407-14-802 PMC4233032

[23] Miyaki, M. et al. Higher frequency of Smad4 gene mutation in human colorectal cancer with distant metastasis. *Oncogene* **18**, 3098–3103. 10.1038/sj.onc.1202642 (1999). 10340381 10.1038/sj.onc.1202642

[24] Chang, S. C. et al. Mutation spectra of common Cancer-Associated genes in different phenotypes of colorectal carcinoma without distant metastasis. *Ann. Surg. Oncol.* **23**, 849–855. 10.1245/s10434-015-4899-z (2016). 26471487 10.1245/s10434-015-4899-z

[25] Duan, L. et al. S100A8 and S100A9 are associated with colorectal carcinoma progression and contribute to colorectal carcinoma cell survival and migration via Wnt/β-catenin pathway. *PLoS One*. **8**, e62092. 10.1371/journal.pone.0062092 (2013). 23637971 10.1371/journal.pone.0062092 PMC3637369

[26] Li, S. et al. S100A8 promotes epithelial-mesenchymal transition and metastasis under TGF-β/USF2 axis in colorectal cancer. *Cancer Commun. (Lond)*. **41**, 154–170. 10.1002/cac2.12130 (2021). 33389821 10.1002/cac2.12130 PMC7896751

[27] Fujita, Y. et al. Regulation of S100A8 stability by RNF5 in intestinal epithelial cells determines intestinal inflammation and severity of colitis. *Cell. Rep.* **24**, 3296–3311e3296. 10.1016/j.celrep.2018.08.057 (2018). 30232010 10.1016/j.celrep.2018.08.057 PMC6185744

[28] Ansari, I. et al. The microbiota programs DNA methylation to control intestinal homeostasis and inflammation. *Nat. Microbiol.* **5**, 610–619. 10.1038/s41564-019-0659-3 (2020). 32015497 10.1038/s41564-019-0659-3

[29] Woo, V. & Alenghat, T. Epigenetic regulation by gut microbiota. *Gut Microbes*. **14**, 2022407. 10.1080/19490976.2021.2022407 (2022). 35000562 10.1080/19490976.2021.2022407 PMC8744890

[30] Allen, J. & Sears, C. L. Impact of the gut Microbiome on the genome and epigenome of colon epithelial cells: contributions to colorectal cancer development. *Genome Med.* **11**, 11. 10.1186/s13073-019-0621-2 (2019). 30803449 10.1186/s13073-019-0621-2 PMC6388476

[31] Xu, Y. et al. The Microbiome types of colorectal tissue are potentially associated with the prognosis of patients with colorectal cancer. *Front. Microbiol.* **14**, 1100873. 10.3389/fmicb.2023.1100873 (2023). 37025624 10.3389/fmicb.2023.1100873 PMC10072283

[32] He, T., Cheng, X. & Xing, C. The gut microbial diversity of colon cancer patients and the clinical significance. *Bioengineered* **12**, 7046–7060. 10.1080/21655979.2021.1972077 (2021). 34551683 10.1080/21655979.2021.1972077 PMC8806656

[33] Zhao, L. et al. Characterization of the consensus mucosal Microbiome of colorectal cancer. *NAR Cancer*. **3**10.1093/narcan/zcab049 (2021). 10.1093/narcan/zcab049 PMC8693571 34988460

[34] Mouradov, D. et al. Oncomicrobial community profiling identifies clinicomolecular and prognostic subtypes of colorectal cancer. *Gastroenterology* **165**, 104–120. 10.1053/j.gastro.2023.03.205 (2023). 36933623 10.1053/j.gastro.2023.03.205

[35] Pan, H. W. et al. Biodiversity and richness shifts of mucosa-associated gut microbiota with progression of colorectal cancer. *Res. Microbiol.* **171**, 107–114. 10.1016/j.resmic.2020.01.001 (2020). 31982498 10.1016/j.resmic.2020.01.001

[36] Cuesta, S., Burdisso, P., Segev, A., Kourrich, S. & Sperandio, V. Gut colonization by Proteobacteria alters host metabolism and modulates cocaine neurobehavioral responses. *Cell. Host Microbe*. **30**, 1615–1629e1615. 10.1016/j.chom.2022.09.014 (2022). 36323315 10.1016/j.chom.2022.09.014 PMC9669251

[37] Arthur, J. C. et al. Intestinal inflammation targets Cancer-Inducing activity of the microbiota. *Science* **338**, 120–123. 10.1126/science.1224820 (2012). 22903521 10.1126/science.1224820 PMC3645302

