# Supplementary material for: Integrative multi-omics deciphers the potential mechanism and microbial biomarkers for lymph node metastasis in colorectal cancer
Source: Sci Rep. 2025 Nov 4;15:38611. doi: 10.1038/s41598-025-22350-2 (PMC12586503; doi:10.1038/s41598-025-22350-2)

**SUPPLEMENTARY FIGURE LEGENDS**

**Supplementary Fig. S1** Pie chart demonstrates distribution of KRAS mutation types among CRC patients.

CRC, colorectal cancer.


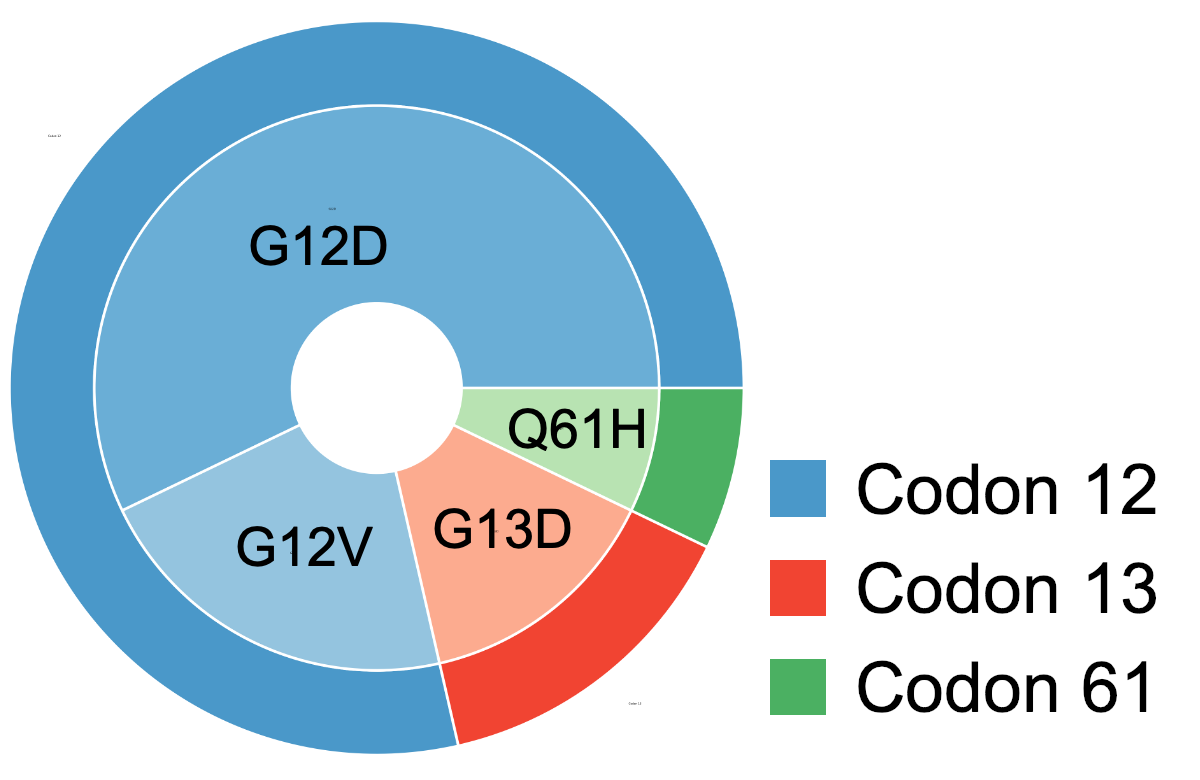


**Supplementary Fig. S2** Dot plot of top 10 enriched pathways determined from GSEA results. Each dot plot demonstrates enriched pathways in CRC tumor (A) and positive LNM (B) comparison of GSEA results. The x-axis is the NES. The size of the dot represents gene count, and the color represents the adjusted *p* value. GO enrichment analysis for genes identified between normal vs. tumor tissues (C); between LNM negative vs. positive (D).

GSEA, gene set enrichment analysis; CRC, colorectal cancer; LNM, lymph node metastasis; NES, normalized enrichment score; GO, gene ontology.


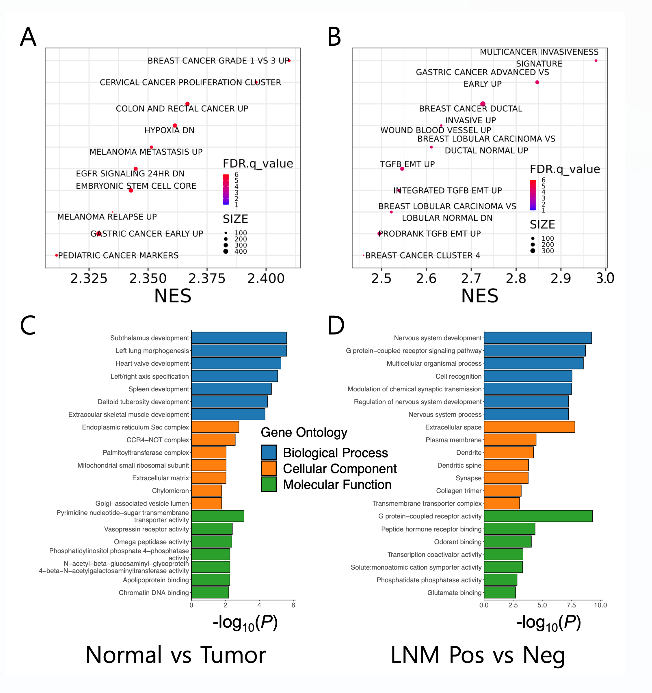


**Supplementary Fig. S3** Heatmap of the differentially methylated genes between tumor and matched normal tissues in CRC (A); between LNM negative vs. positive (B).

CRC, colorectal cancer; LNM, lymph node metastasis.


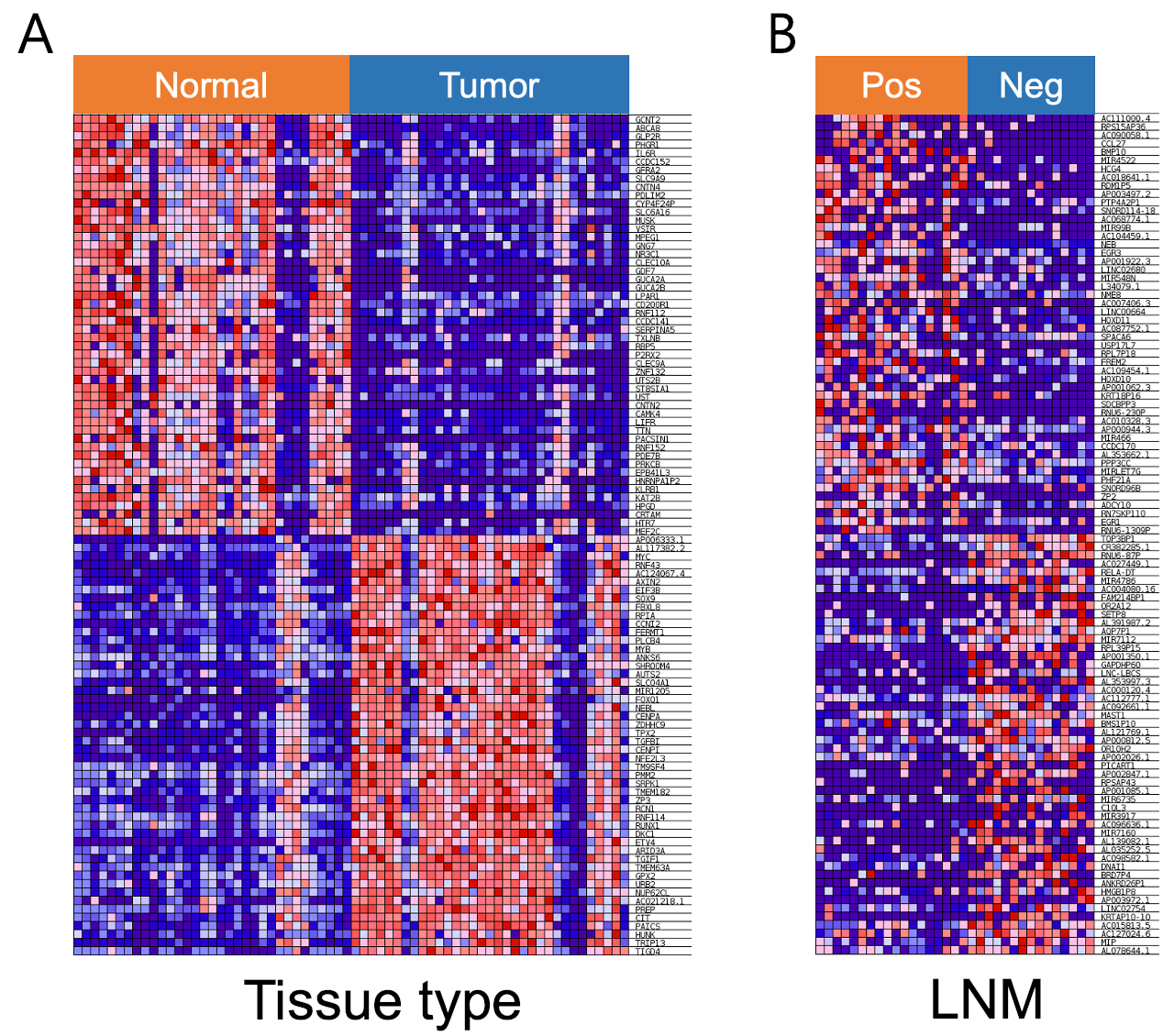


**Supplementary Fig. S4** (A) Comparison of gene set analysis presented as a log-transformed odd ratios provided by CMS subtype. Hierarchical clustering heatmap of DNA methylation profiles shows distinct patterns between CMS2 other CMS groups (B); between CMS4 other CMS groups (C).

CMS, consensus clustering subtypes; DNA, deoxyribonucleic acid.


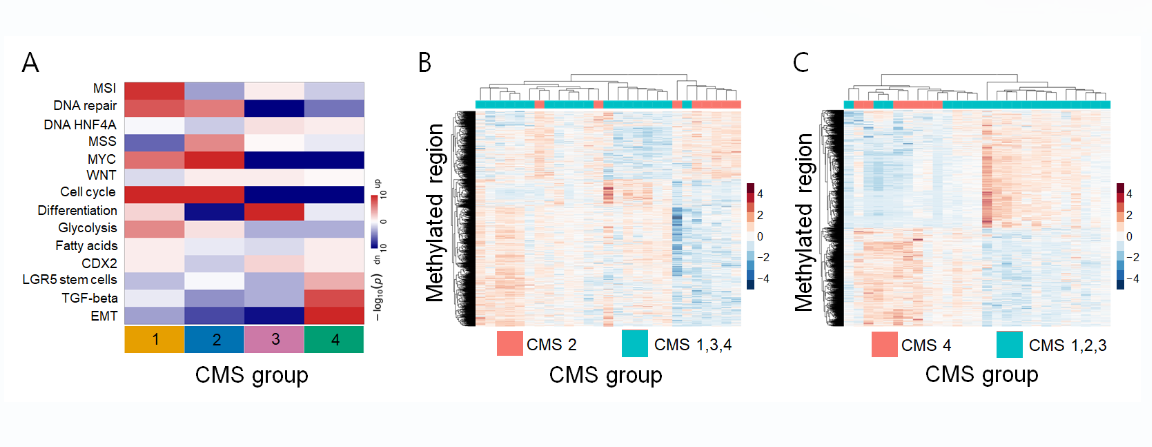


**Supplementary Fig. S5** Transcription factor motif enrichment analysis in promoter region of correlated genes for CMS subtypes in CRC.

CMS, consensus clustering subtypes; CRC, colorectal cancer.


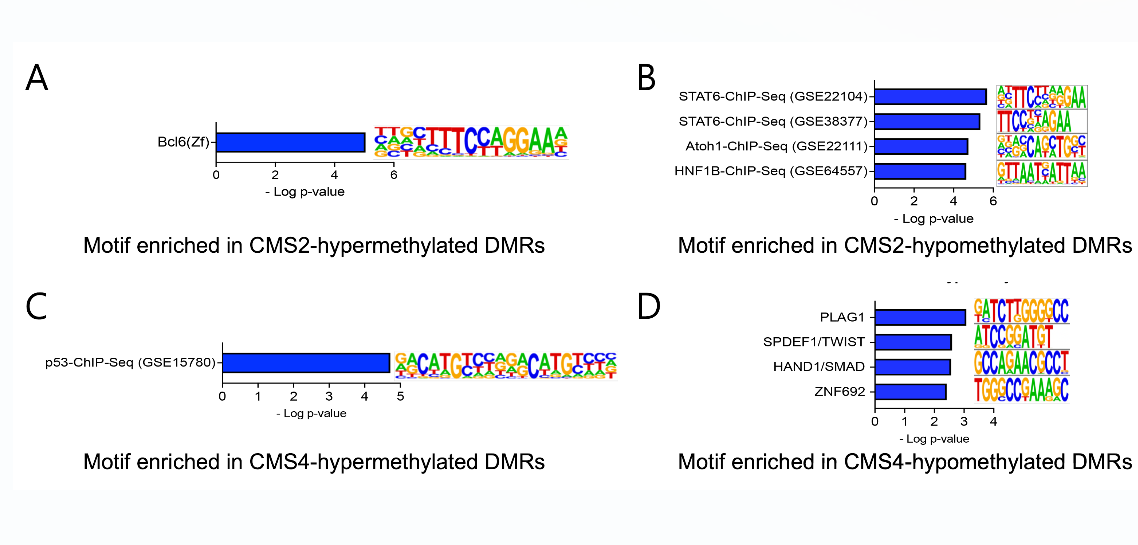


**Supplementary Fig. S6** PCA plot indicates separation of patients with high *p*_PU and low *p*_PU group, with some overlap between them.

PCA, principal component analysis; *p*_PU, *phylum* Proteobacteria unassigned.


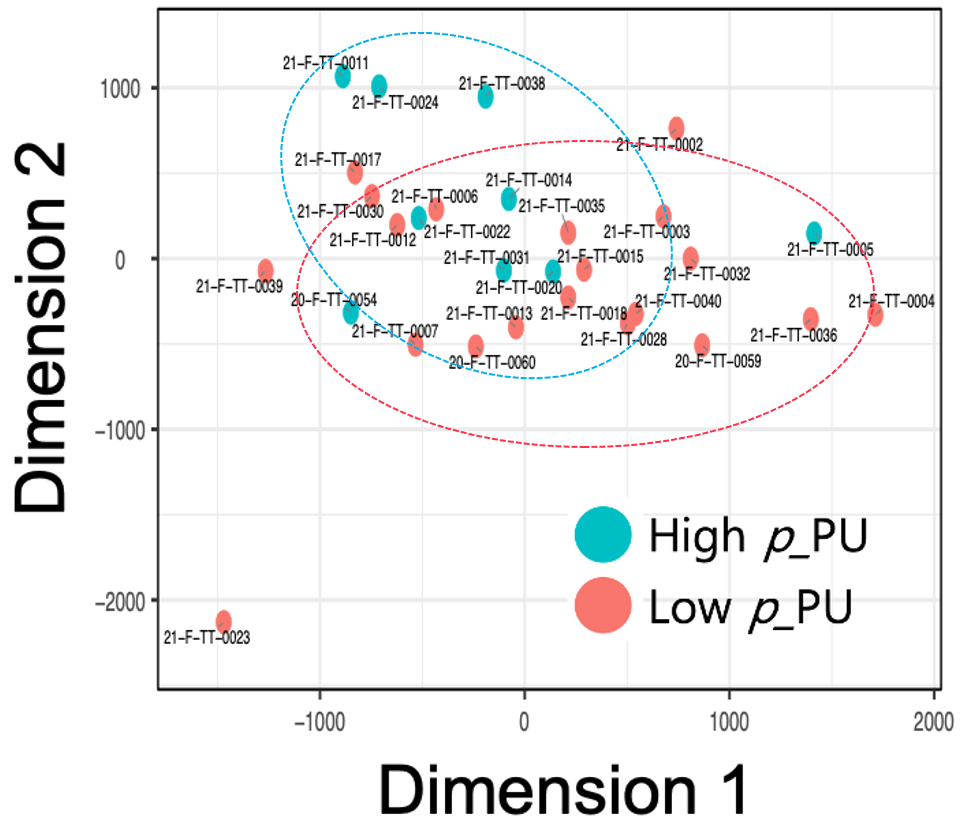

Supplement: Supplementary file 1 — Supplementary Material 1 [file 41598_2025_22350_MOESM1_ESM.docx]
